# Olfactory Function as a Potential Predictor of Cognitive Impairment in Men and Women

**DOI:** 10.3390/biology13070503

**Published:** 2024-07-05

**Authors:** Carla Masala, Francesco Loy, Ilenia Pinna, Nicoletta Aurora Manis, Tommaso Ercoli, Paolo Solla

**Affiliations:** 1 Department of Biomedical Sciences, University of Cagliari, SP 8 Cittadella Universitaria, 09042 Monserrato, Italy; floy@unica.it (F.L.); ilenia.pinna.1994@gmail.com (I.P.); nicolettaamanis@gmail.com (N.A.M.); 2 Neurological Unit, AOU Sassari, University of Sassari, Viale S. Pietro 10, 07100 Sassari, Italy; ercolitommaso@me.com (T.E.); psolla@uniss.it (P.S.)

**Keywords:** cognitive abilities, healthy subjects, olfactory function, Montreal Cognitive Assessment, aging

## Abstract

**Simple Summary:**

The aim of this study was to analyze the role of each factor of the olfactory function as a predictor of cognitive impairment in relation to gender and age. Our results indicated that in men significant correlations were found in odor threshold versus language index score, as well as in odor identification versus language and executive index score. Instead, in women, odor discrimination and identification were related to visuospatial index score.

**Abstract:**

Background: Different previous studies indicated olfactory function as a predictor of several types of cognitive impairment, in particular related to neurodegenerative disease. However, scanty data are available on the role of odor threshold (OT), odor discrimination (OD), and odor identification (OI) as a predictor of cognitive impairment. The aim of this study was to evaluate potential correlations between each factor of the olfactory function versus each specific cognitive domain of the Montreal Cognitive Assessment (MoCA) test on healthy subjects in relation to gender and age. Methods: Sniffin’ Sticks and MoCA tests were used to determine olfactory function and cognitive abilities, respectively. Results: In men, significant correlations were found in OT versus language index score and OI versus language and executive index score, while in women, OD and OI were correlated to visuospatial index score. Conclusions: Our data suggested that olfactory function (OT, OD, and OI) may be considered a predictor for cognitive impairment in relation to gender and age.

## 1. Introduction

Aging is characterized by progressive muscle mass loss, a decrease in cell turnover, and by an increase in brain atrophy. Usually, the normal process of aging is different from pathologic changes that occur more drastic due to the impairment of compensatory mechanisms. Impaired compensatory mechanisms predispose individuals to cognitive dysfunction, mild cognitive impairment (MCI), dementia, and neurodegenerative diseases [1,2,3,4].

Cognitive function is affected by numerous factors that could cause problems in several aspects of life, including memory, executive function, ability, work, social activities, etc. Indeed, cognitive decline has been already related to age, gender, obesity, level of education, psychophysical activity, and social activity [5]. From this perspective, MCI was estimated to have a relevant increase annually [6] and was associated with different pathologies. Unfortunately, spotting the signs of cognitive decline is difficult since symptoms take a long time to become known and diagnosis may be delayed. Moreover, aging is related to a decrease in cognitive performance with an irregular manner that increases rapidly after middle-age.

Olfactory function has been investigated as a potential biomarker in MCI and neurodegenerative conditions, including Alzheimer’s disease (AD) and Parkinson’s disease (PD) [7,8,9,10,11]. In particular, Jung and colleagues [8] in a meta-analysis indicated that odor identification is impaired in patients with AD and MCI, and this olfactory deficit appears more severe in AD than in MCI. Moreover, olfactory impairment may usually precede the appearance of clinical motor symptoms in PD patients, as indicated in previous studies [10,11], and is supportive criteria for PD diagnosis according to the Clinical Diagnostic Criteria for Parkinson’s Disease.

The decrease in olfactory perception is a process related to aging and is therefore a physiological process. However, olfactory dysfunction (hyposmia) has been correlated with neurodegenerative diseases. For this reason, it is essential to distinguish between physiological processes and pathological ones to ensure a better diagnosis for the patient [4,12]. Different previous studies suggested that alterations in odor identification may precede cognitive decline and may be considered as biomarkers in elderly subjects [13,14]. In addition, the olfactory deficit may be a predictor for the transition from MCI to AD.

Some studies reported gender differences in olfactory function. Indeed, it has been reported that women frequently exhibited better olfactory acuity compared to men, probably due to hormonal fluctuations, genetic predispositions, and neuroanatomical variances [15,16].

The pathways that underlie the association between olfactory dysfunction and MCI remain incompletely elucidated. Current knowledge indicates that both olfactory dysfunction and cognitive decline might be interconnected with common pathological mechanisms, involving neurodegenerative processes, inflammatory responses, and cerebrovascular alterations [3,17]. The evaluation of olfactory function is a non-invasive and economically feasible screening tool to identify individuals predisposed to cognitive decline and dementia. An early diagnosis of any neurodegenerative disease may improve the patient’s quality of life and potential pharmacological treatment [18]. Based on these considerations, the aim of this study was to evaluate potential correlations between each factor of the olfactory function, such as odor threshold (OT), odor discrimination (OD), and odor identification (OI), versus each specific cognitive domain of the Montreal Cognitive Assessment (MoCA) test on healthy subjects in relation to gender and age.

## 2. Materials and Methods

### 2.1. Participants

In this research, 339 healthy subjects (220 women and 119 men) with a mean age of 39.9 ± 18.5 years were consecutively enrolled. Participants were recruited at the Department of Biomedical Sciences at the University of Cagliari, starting from February 2017 to November 2022. Inclusion criteria were participants with an age range from 18 to 85 years.

Subjects were divided in two age groups: 18–35 years (*n* = 122 and *n* = 60 in women and men, respectively) and 36–> 55 years (*n* = 98 and *n* = 59 in women and men, respectively). Exclusion criteria were upper respiratory infections, asthma, neurodegenerative diseases, a history of head or neck trauma, stroke, diabetes, and any systemic disease associated with olfactory and gustatory disorders. In all subjects were collected age, height (m), weight (kg), body mass index (BMI, kg/m^2^), olfactory and gustatory functions, smoking habits, formal education (>12 years of formal education), and cognitive abilities.

The study was conducted in accordance with the Declaration of Helsinki and approved by the Ethics Committee of the Azienda Ospedaliero Universitaria (A.O.U.), University of Cagliari (PROT. NP/2018/1630). All experimental procedures were explained to participants, who signed the informed consent before the start of the experiment.

### 2.2. Procedures for Olfactory Function Evaluation

The olfactory function was assessed by the Sniffin’ Sticks test, which is a validated tool with three different sub-tests: OT, OD, and OI [19,20]. First, the OT was evaluated using the n-butanol of 16 stepwise dilutions. The OT was also assessed using a single-staircase technique based on the three-alternative forced choice (3AFC) task. Second, in the OD test, three pens were presented, two containing the same odor and the third containing the target odorant using the 3AFC task. Third, the OI was evaluated using 16 common odors, each presented with four verbal descriptors in a multiple forced-choice format (three distractors and one target). Total scores (threshold + discrimination + identification = TDI) were calculated. TDI scores of ≤16 between 16.25 and 30.5, between 30.75 and 41.25, and >41.5 were indicated as functional anosmia, hyposmia, normosmia, and supersmellers, respectively [21]. In each participant, olfactory tests, gustatory assessment, and cognitive abilities were performed during the same session in a well-ventilated room. First, were evaluated olfactory function, then cognitive abilities, and finally gustatory function.

### 2.3. Procedures for Gustatory Function Evaluation

The gustatory function was assessed using the “Taste Strips” test (Burghart Messtechnik, Wedel, Germany). The “Taste Strips” test consists of filter paper strips impregnated with four concentrations of each basic taste quality: sweet, bitter, sour, and salty [22]. The “Taste Strips” test concentrations were the following: for sweet taste 0.4, 0.2, 0.1, 0.05 g/mL of sucrose; for bitter taste 0.006, 0.0024, 0.0009, and 0.0004 g/mL of quinine hydrochloride; for sour 0.3, 0.165, 0.09, and 0.05 g/mL of citric acid; and for salty taste 0.25, 0.1, 0.04, and 0.016 g/mL of sodium chloride [22,23]. Drinking water has been used as a solvent after each taste modality and to wash the participant’s mouth before each taste stimuli. Total “Taste Strips” score may range from 0 to 16, and a taste score ≥9 was considered normogeusia, and a score of <9 was classified as hypogeusia [22,23].

### 2.4. Procedures for Cognitive Abilities Evaluation

Cognitive abilities were measured by the Montreal Cognitive Assessment (MoCA), which assesses potential cognitive impairment in different domains: visual-constructional skills, executive functions, attention and concentration, memory, language, conceptual thinking, calculations, and spatial orientation [24,25]. The total score in the MoCA test was 30, and any score of ≥26 was considered normal. In the MoCA test, the following 6 index sub-scores of cognitive function-specific domains were determined according to the study of Julayanont and colleagues [26]: Orientation Index Score (OIS), Attention Index Score (AIS), Language Index Score (LIS), Visuospatial Index Score (VIS), Memory Index Score (MIS), and Executive Index Score (EIS).

### 2.5. Statistical Analyses

The simple size calculation was carried out to assess the adequate minimum of participants to enroll. Considering previous studies with similar topics [1,11], about 300 participants could be suitable to detect significant differences. Similarly, 250 participants were indicated by the power calculation, considering a critical effect size of medium level (f = 0.15–0.25) with 95% power and the 5% of significance level in the two-way ANOVA. The Shapiro–Wilk test for normality was carried out.

Statistical analyses were carried out using SPSS 26.0 for Windows (IBM, Armonk, NY, USA). All data were indicated as mean values ± standard deviation (SD). The two-way ANOVA has been performed to evaluate the effect of gender and age on olfactory function (OT, OD, OI, and TDI score) and cognitive abilities (OIS, AIS, LIS, VIS, MIS, and EIS). Post hoc analyses, using multiple pairwise comparison tests with Bonferroni’s corrected alpha values, were achieved to calculate statistical differences. Considering the percentages of smokers and subjects with >12 years of formal education, statistical differences were performed using the Chi-Square test (Χ^2^).

Bivariate correlations between each specific domain of cognitive function (OIS, AIS, LIS, VIS, MIS, and EIS) and each sub-test of olfactory function (OT, OD, and OI) were performed using Pearson’s correlation coefficient (r) to determine more promising factors for multiple linear regression analysis. Multiple linear regression analyses were computed in three different models using each sub-test of olfactory function (OT, OD, and OI) as a dependent variable, while each specific domain of cognitive function (OIS, AIS, LIS, VIS, MIS, and EIS) was considered an independent variable. Significant level was set at 0.05.

## 3. Results

### Descriptive Statistics of the Subjects

In our sample, we observed a statistically significant interaction of gender and age on weight [F_(1,335)_ = 3.52, *p* < 0.05, partial η^2^ = 0.021], height [F_(1,335)_ = 6.86, *p* < 0.001, partial η^2^ = 0.039], and BMI [F_(1,335)_ = 11.1, *p* < 0.0001, partial η^2^ = 0.062]. Post hoc test analyses showed that men exhibited a significant increase in weight, height, and BMI than women in the two ages ranges (18–35 and 36–> 55 years). In women, we observed that smokers were 27.8% (*n* = 34) and 26.5% (*n* = 26) in the age ranges 18–35 and 36–> 55 years, respectively. In men, smokers were 41.7% (*n* = 25) and 47.5% (*n* = 28) in the age ranges 18–35 and 36–> 55 years, respectively. Most women and men in the two age ranges showed >12 years of formal education, as indicated in Table 1. No significant differences were observed between men and women for % of smokers and formal education in all age ranges (Table 1).

Considering olfactory function, a two-way ANOVA was performed to examine the effect of gender and age on OT, OI, OD, and TDI score. A statistically significant interaction between the effect of gender and age was observed only for OT [F_(1,335)_ = 5.03, *p* < 0.01, partial η^2^ = 0.029], OD [F_(1,335)_ = 4.83, *p* < 0.01, partial η^2^ = 0.028], and TDI score [F_(1,335)_ = 5.03, *p* < 0.01, partial η^2^ = 0.031]. Post hoc test analyses showed that in men, a significant decrease in olfactory perception (OT) between subjects with 18–35 years and 36–> 55 years was observed. Instead, in women, a significant decrease (*p* < 0.05) in OD scores between 36 and >55 versus 18 and 35 years old age ranges was found. Moreover, significant differences (*p* < 0.05) between the 36 and >55 and 18 and 35 years age ranges, both in women and in men, were observed. No significant differences were found for OI (Table 2).

Among women, in the age range 18–35 years, 73.8% (*n* = 90), 25.4% (*n* = 31), and 0.8% (*n* = 1) showed normosmia, hyposmia, and anosmia, respectively. Indeed, in the age range 36–> 55 years, 65.3% (*n* = 64), 29.6% (*n* = 29), and 5.1% (*n* = 5), for normosmia, hyposmia, and anosmia, respectively. In men, in the age range 18–35 years, 70% (*n* = 42), 28.3% (*n* = 17), and 1.7% (*n* = 1) men in the age range 18–35 years showed normosmia, hyposmia, and anosmia, respectively; while, in the age range 36–> 55 years, 65.3% (*n* = 64), 29.6% (*n* = 29), and 5.1% (*n* = 5) showed normosmia, hyposmia, and anosmia, respectively.

Similarly, we found a statistically significant interaction between the effects of gender and age on MIS [F_(1,335)_ = 10.23, *p* < 0.0001, partial η^2^ = 0.058]. Post hoc test analyses showed a significant decrease (*p* < 0.01) in MIS score was observed between the two age ranges in women, and significant differences (*p <* 0.001) were also observed between the age range 18–35 years in women versus 36–> 55 years in men (Table 2). In women, a MoCA total score of ≤26 was observed in 22.9% (*n* = 28) and 33.7% (*n* = 33) in two age ranges, respectively. In men, a MoCA total score of ≤26 has been observed in 26.6% (*n* = 16) and 43.4% (*n* = 25) in 18–35 and 36–> 55 age groups, respectively.

Pearson’s correlations were performed to evaluate associations between each factor of olfactory function and each index sub-score of cognitive function in men and women (Table 3). Table 3 indicated the following significant positive correlations in men: between OT versus LIS (*p* < 0.01), OI versus LIS, and EIS (*p* < 0.01).

Table 4 indicated Pearson correlations (r) between each factor of the olfactory function and each index sub-score of cognitive function in women. No significant correlations were found between OT and each index sub-score of cognitive function in women. Instead, a significant positive correlation (*p* < 0.05) was observed only between VIS versus OD and OI.

**Figure 1:**
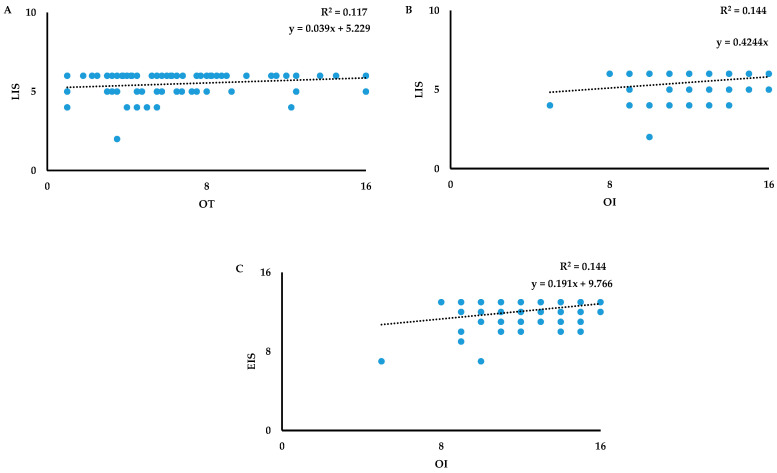
Scatterplots of the relationship between odor threshold (OT) versus Language Index Score (LIS) (**A**), between odor identification (OI) versus LIS (**B**), and between OI versus Executive Index Score (EIS) (**C**) in men.

Moreover, to better explain the effect of bivariate Pearson’s correlations, multiple regression analyses were performed to predict olfactory dysfunction in men and women in relation to each specific index sub-score of cognitive abilities. In men, only LIS was a significant predictor of the OT function [F_(1,118)_ = 9.302, *p* < 0.01] with a model that explained 12% of the variance (Table 5, Figure 1A). Whereas, using OI as a dependent variable, LIS (Figure 1B) and EIS (Figure 1C) sub-scores of cognitive abilities were significant predictors for the OI deficits [F_(1,118)_ = 6.469, *p* < 0.001] with a model that explained 14.4% of the variance. Instead, no significant associations were observed between each index sub-score of the MoCA test and OD in men.

**Figure 2:**
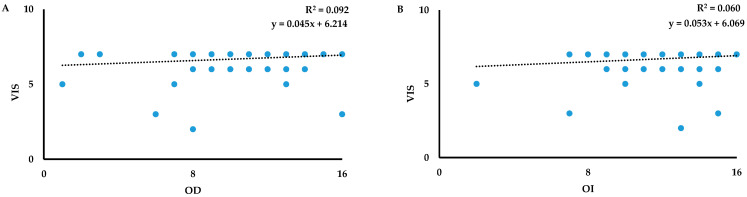
Scatterplots of the relationship between: (**A**) odor discrimination (OD) versus Visuospatial Index Score (VIS) and (**B**) odor identification (OI) versus Executive Index Score (EIS) in women.

In women, no significant associations were observed between each index sub-score of the MoCA test and OT (Table 6). Instead, VIS was a significant predictor of OD [F_(1,218)_ = 11.038, *p* < 0.001] and OI [F_(1,217)_ = 6.775, *p* < 0.001] with a model that explained 9.2% (Figure 2A) and 6% (Figure 2B) of the variance, respectively.

## 4. Discussion

Olfactory function and cognitive abilities usually decrease in relation to age [27,28]. Our study, for the first time, focused on the evaluation of potential correlations between each parameter of the olfactory function (OT, OD, and OI) versus each specific cognitive domain of the MoCA test (AIS, EIS, LIS, MIS, OIS, and VIS) on healthy participants in relation to gender and age to facilitate an early detection of mild cognitive impairment. Other previous studies evaluated correlations between olfactory and cognitive functions, focusing on elderly subjects with cognitive impairment and dementia [29,30,31,32,33]. In our study, considering olfactory function, using the two-way ANOVA, we found statistically significant interactions between the effect of gender and age only for OT, OD, and TDI score. Post hoc test analyses showed that in men, a significant decrease in OT between subjects with 18–35 years and 36–> 55 years. Instead, in women, a similar significant decrease in OD scores between the age ranges 18–35 years versus 36–> 55 years was found. Moreover, as regards cognitive abilities, a statistically significant interaction between effects of gender and age on MIS was found.

The differences in memory domain could be related to gender; it is known that verbal memory and spatial memory tasks are better addressed by women than men. A recent study showed that gender was a predictor for the MoCA-MIS, and women had higher scores than men [34]. Although the authors did not evaluate olfactory function in their study, their results support our findings for cognitive abilities. Moreover, other studies showed a significant increase in MIS score in women than men [35,36]. Gender differences in MIS may also reflect changes in functional brain organization. This could be explained considering that women may integrate pathways from different neural networks due to the beneficial effects of estrogens on hippocampal neuronal activity [35]. Moreover, women exhibited larger hippocampal brain volume than men [37]. Instead, another study suggested that men and women exhibited similar memory abilities in elderly subjects [38].

As regards associations between olfactory function and cognitive abilities in men, our study showed significant correlations in OT versus LIS and in OI versus LIS and EIS. The OT is related to individual differences of the nasal cavity and the nasal epithelium [39], while OI and OD are more related to the central nervous system such as the piriform cortex and the orbitofrontal cortex [40,41,42].

The OI is an easy objective test for clinical evaluation, which has become increasingly important in establishing cognitive abilities in patients, especially in relation to aging or to different neurodegenerative disorders. Some authors obtained contradictory results on gender differences in OI, but usually a better OI was observed in women [43,44,45,46]. A recent study on elderly people showed that executive function was correlated to OI in men [47], similar to our results. Regarding visuospatial ability, Zhong and colleagues indicated a correlation with OI in men and women, while we found the same correlation only in women. Moreover, authors indicated that the correlation between cognitive domains and OI was stronger in women, except for working memory [47]. These observed differences between men and women may be due to different influences, such as neuroendocrine and hormonal factors and fluctuations of the menstrual cycle, but also previous experiences, cultural practices, and dietary behavior. In addition, it is important to note that morpho-anatomical characteristics such as intranasal volume [48] or the expression of receptors in the nasal mucosa [49] did not show significant differences in relation to gender. Moreover, the OI scores are strongly associated with cultural differences; in fact, in our previous study, the OI scores in Sardinia were significantly higher than those in other Italian regions. Within this context, a significant decrease in OI scores was observed in people with >60 years in both men and women [45].

Some authors have also correlated Language to OI [50,51,52]. Two previous studies [51,52] suggested that higher scores in verbal skills were associated with better OI scores. Since OI is considered the ability to recognize, distinguish, and name an odor, the findings may be explained considering a common pathway between OI and verbal skills, including the right insular cortex and the lateral temporal lobe [51].

Westervelt and colleagues showed in their cohort of men and women that the Language domain was a significant predictor of OI, suggesting a specific role of the temporal limbic process [50]. Our results showed that LIS was correlated to OT and OI in men; however, it is difficult to evaluate the specific causal relationship between these factors. Moreover, our previous study associated OT with specific cognitive domains, such as language function in Parkinson’s disease patients [53].

In women, our results demonstrated that OD and OI were correlated to VIS. Similarly, other authors observed a significant correlation between OI and visuospatial skills in men and women [50]. A previous study reported associations between olfactory identification and executive functioning [54]. These results may suggest an overlapping between olfactory brain areas and executive functioning with the integrity of the orbitofrontal cortex. The integrity of orbitofrontal cortex is usually involved in reward behavioral response, decision-making, and working memory. Although this study is performed in a large population of healthy subjects, a limitation should be indicated.

Our study showed some limitations since it was designed as a cross-sectional analysis and did not report longitudinal data and bioimaging analyses. Our sample was enrolled in a specific country, so it should be suitable to compare our data with similar ones obtained in other countries. A multicentric approach could be the next step of our research. Further studies are needed to better understand the nature and mechanism of the correlations between olfactory function and cognitive abilities in relation to gender.

## 5. Conclusions

This study supports the hypothesis that olfactory function (OT, OD, and OI) may be considered a predictor for cognitive impairment in relation to gender and age.

A significant decrease in OT between the two age ranges was observed in men, while a similar significant decrease was found in women for OD score. As regard to cognitive abilities, a statistically significant interaction between effects of gender and age on MIS was found.

Our data suggested that in men, OT and OI were predictors for LIS; in addition, OI was also the predictor for EIS. Instead, in women, OD and OI were predictors for VIS. This study suggests that olfactory tests in combination with other cognitive tests may contribute to the quantity of the risk of cognitive decline during aging. Olfactory evaluations could highlight an early diagnosis of cognitive impairment to promote new personalized therapeutic approaches.

## Figures and Tables

**Table 1:** Demographic information in men and women in relation to different age ranges.

	Women		Men			
Parameters	18–35	36–> 55	18–35	36–> 55	*p*	Post Hoc
Weight	57.5 ± 9.8	60.9 ± 10.5	73 ± 12.9	75.4 ± 10.3	**0.031**	a; b; c
Height	1.6 ± 0.1	1.5 ± 0.1	1.7 ± 0.1	1.7 ± 0.1	**0.001**	d; e; f
BMI	22.1 ± 3.3	24.2 ± 4.2	24 ± 4.1	25.5 ± 3.3	**0.0001**	g; h; i
Smokers (%)	27.8%	26.5%	41.7%	47.5%	>0.05	
>12 years of formal education	87.1%	71.4%	86.6%	62.7%	>0.05	

Legend: Significant *p* values are highlighted in bold. Data are expressed as mean value ± standard deviation; a = *p* < 0.001 between 18 and 35 (women) versus 18 and 35 years (men); b = *p* < 0.001 between 36 and >55 (women) versus 18 and 35 years (men); c = *p* < 0.001 between 18 and 35 (women) versus 36 and >55 years (men); d = *p* < 0.05 in women between 18 and 35 versus 36 and >55 years; e = *p* < 0.001 between 18 and 35 (women) versus 18 and 35 years (men); f = *p* < 0.001 between 18 and 35 (women) versus 36 and >55 years (men); g = *p* < 0.0001 in women between 18 and 35 versus 36 and >55 years; h = *p* < 0.05 between 18 and 35 (women) versus 18 and 35 years (men); i = *p* < 0.0001 between 18 and 35 (women) versus 36 and >55 years (men); statistical differences for weight, height, and BMI were carried out using two-way ANOVA followed by Bonferroni’s post hoc test, while statistical differences for percentages of smokers and >12 years of formal education were performed using Chi-Square test (Χ^2^).

**Table 2:** Clinical information for olfactory function and cognitive abilities in men and women in relation to age.

	Women		Men			
Parameters	18–35	36–> 55	18–35	36–> 55	*p*	Post Hoc
OT	7.6 ± 4.2	7.3 ± 4.4	8.6 ± 4.9	6.1 ± 4.2	**0.007**	j
OD	12.2 ± 1.8	11.3 ± 2.6	11.4 ± 2.3	11.4 ± 2.3	**0.009**	k
OI	13.1 ± 1.5	12.7 ± 2.3	13 ± 1.7	12.6 ± 2.2	0.172	
TDI Score	32.9 ± 5	31.3 ± 7.1	33 ± 6.7	30.1 ± 6.3	**0.005**	l
OIS	6 ± 0.01	5.9 ± 0.1	6 ± 0.01	6 ± 0.01	0.385	
AIS	8.4 ± 0.9	8.3 ± 1	8.5 ± 0.8	8.3 ± 1.1	0.251	
LIS	5.5 ± 0.7	5.6 ± 0.7	5.6 ± 0.7	5.5 ± 0.7	0.317	
VIS	6.8 ± 0.7	6.8 ± 0.6	6.7 ± 0.8	6.6 ± 0.7	0.623	
MIS	3.7 ± 1.3	2.9 ± 1.5	3.3 ± 1.4	2.6 ± 1.7	**0.0001**	m; n
EIS	12.3 ± 1.1	12.2 ± 1.1	12.4 ± 1.1	12 ± 1.2	0.161	

Legend: Data are expressed as mean value ± standard deviation. OT—odor threshold; OD—odor discrimination; OI—odor identification; TDI—threshold + discrimination + identification. Data are expressed as mean value ± standard deviation. OIS—Orientation Index Score; AIS—Attention Index Score; LIS—Language Index Score; VIS—Visuospatial Index Score; MIS—Memory Index Score; EIS—Executive Index Score. The *p* values < 0.05 are highlighted in bold; j = *p* < 0.05 in men between 18 and 35 versus 36 and >55; k = *p* < 0.05 in women between 18 and 35 versus 36 and >55 years; l = *p* < 0.05 between 18 and 35 (women) versus 36 and >55 years (men); m = *p* < 0.01 between 18 and 35 versus 36 and >55 years in women; n = *p* < 0.001 between 18 and 35 (women) versus 36 and >55 years (men); statistical differences were carried out using two-way ANOVA followed by Bonferroni’s post hoc test.

**Table 3:** Pearson’s correlations (r) between olfactory function and each index sub-score of MoCA representative of specific domains of cognitive function in men.

Parameters	OT	OD	OI
OIS	0.011	−0.023	−0.105
AIS	0.157	0.064	0.165
LIS	0.259 **	0.084	0.240 **
VIS	0.158	0.149	0.310
MIS	0.052	0.101	0.147
EIS	0.018	0.152	0.324 **

Legend: OIS—Orientation Index Score; AIS—Attention Index Score; LIS—Language Index Score; VIS—Visuospatial Index Score; MIS—Memory Index Score; EIS—Executive Index Score; OT—odor threshold; OD—odor discrimination; OI—odor identification. ** = *p* < 0.01.

**Table 4:** Pearson’s correlations (r) between olfactory function and each index sub-score of cognitive function in women.

Parameters	OT	OD	OI
OIS	−0.053	0.054	−0.110
AIS	0.056	0.034	0.034
LIS	0.094	0.045	0.047
VIS	0.037	0.157 *	0.154 *
MIS	−0.011	0.089	0.002
EIS	0.059	0.048	0.093

Legend: AIS—Attention Index Score; EIS—Executive Index Score; LIS—Language Index Score; MIS—Memory Index Score; OIS—Orientation Index Score; VIS—Visuospatial Index Score; OT—odor threshold; OD—odor discrimination; OI—odor identification. * = *p* < 0.05.

**Table 5:** Multiple regression analysis models performed using each factor of the olfactory function and each index sub-score of cognitive function in men.

Parameters	Unstandardized Coefficients		Standardized Coefficients		
	B	Std Error	β	t	*p*
Model 1: OT as a dependent variable					
Age	−0.057	0.022	−0.229	−2.568	**0.011**
LIS	1.389	0.579	0.214	2.399	**0.018**
Model 2: OD as a dependent variable					
Age	−0.096	−0.967	0.337	−0.091	0.872
OIS	−0.505	2.355	−0.020	−0.214	0.831
AIS	−0.192	0.396	−0.079	−0.485	0.629
LIS	−0.144	0.465	−0.046	−0.310	0.757
VIS	0.168	0.380	0.056	0.441	0.660
MIS	0.115	0.143	0.080	0.805	0.422
EIS	0.377	0.363	0.191	1.039	0.301
Model 3: OI as a dependent variable					
Age	−0.021	0.009	−0.205	−2.291	**0.024**
LIS	0.519	0.242	0.192	2.146	**0.034**
EIS	0.434	0.211	0.256	2.054	**0.042**

Legend: AIS—Attention Index Score; EIS—Executive Index Score; LIS—Language Index Score; MIS—Memory Index Score; OIS—Orientation Index Score; VIS—Visuospatial Index Score; OT—odor threshold; OD—odor discrimination; OI—odor identification; B—unstandardized coefficient for each predictor variable; β—standardized coefficient which gives a measure of the variable contribution; t—t-values which indicate whether the predictor’s regression coefficient is significant. Significant *p* values are indicated in bold.

**Table 6:** Multiple regression analysis models using each parameter of olfactory function and each index sub-score of MoCA representative of specific domains of cognitive function in women.

Parameters	Unstandardized Coefficients		Standardized Coefficients		
	B	Std Error	β	t	*p*
Model 1: OT as a dependent variable					
Age	−0.028	0.017	−0.119	−1.660	0.098
OIS	−3.386	4.428	−0.053	−0.765	0.445
AIS	−0.055	0.506	−0.013	−0.108	0.914
LIS	0.728	0.612	0.123	1.189	0.236
VIS	−0.121	0.630	−0.018	−0.193	0.847
MIS	−0.234	0.241	−0.078	−0.969	0.334
EIS	0.075	0.489	0.020	0.153	0.878
Model 2: OD as a dependent variable					
Age	−0.032	0.008	−2.260	−4.030	**0.0001**
VIS	0.524	0.223	0.152	2.345	**0.020**
Model 3: OI as a dependent variable					
Age	−0.019	0.007	−0.188	−2.847	**0.005**
VIS	0.437	0.192	0.150	2.277	**0.024**

Legend: AIS—Attention Index Score; EIS—Executive Index Score; LIS—Language Index Score; MIS—Memory Index Score; OIS—Orientation Index Score; VIS—Visuospatial Index Score; OT—odor threshold; OD—odor discrimination; OI—odor identification; B—standardized coefficient which gives a measure of the variable contribution; β—standardized coefficient which gives a measure of the variable contribution; t—t-values which indicate whether the predictor’s regression coefficient is significant. Significant *p* values are indicated in bold.

## Data Availability

Data supporting the findings presented in this study will be made available upon reasonable request to the corresponding authors.
